# Longitudinal transitions of cigarettes and electronic nicotine delivery systems among adolescents: Construction of a retrospective cohort using recall data from a cross-sectional sample

**DOI:** 10.18332/tid/128488

**Published:** 2020-11-05

**Authors:** Heewon Kang, Sung-il Cho

**Affiliations:** 1 Institute of Health and Environment, Seoul National University, Seoul, Republic of Korea; 2 Department of Public Health Science, Graduate School of Public Health, Seoul National University, Seoul, Republic of Korea

**Keywords:** adolescents, non-cigarette tobacco/nicotine products, longitudinal transitions, gateway effect

## Abstract

**INTRODUCTION:**

A major concern regarding non-cigarette tobacco or nicotine products (NCTNPs) is whether they facilitate or mitigate overall tobacco or nicotine use. We examined longitudinal transitions of cigarettes and electronic nicotine delivery systems (ENDS) by constructing a retrospective cohort based on the recall data of a cross-sectional sample.

**METHODS:**

Using the Korea Youth Risk Behavior Survey, we constructed crosssectional data of 59576 adolescents into retrospective cohort data. Participants were categorized into 4 mutually exclusive tobacco or nicotine use states. We used a multistate Markov model to identify transitions between the states to calculate transition intensity ratios (TIRs), and examined the current use of tobacco or nicotine products to assess both gateway effects to cigarettes, and whether ENDS use helps adolescents quit cigarette smoking.

**RESULTS:**

Compared with never use, use of ENDS was associated with an increased risk of initiation of cigarette use (TIR=6.8; 95% CI: 4.5–10.2). The risk of transitioning from cigarette ever use to ENDS, compared with never use to ENDS, was even more pronounced (TIR=44.1; 95% CI: 34.1–56.9). The prevalence of current cigarette smoking was higher among those who started ENDS then cigarettes, compared to those who began cigarette use without experimenting with ENDS (43.1% vs 35.8%). Moreover, 27.8% (95% CI: 23.6–32.0%) of adolescents who experimented first with cigarettes then moved to ENDS were current users of cigarettes, and 46.4% (95% CI: 42.1–51.1%) of these adolescents were current users of both cigarettes and ENDS.

**CONCLUSIONS:**

Based on the recall data of a cross-sectional sample, we demonstrate that ENDS experimentation increases the likelihood of cigarette smoking initiation. A significant proportion of these adolescents continue to use cigarettes. Moreover, those who experimented with cigarettes then ENDS also continue smoking cigarettes or both cigarettes and ENDS. We suggest comprehensive tobacco control policies for all tobacco/nicotine products and monitoring the timing of NCTNP initiation in cross-sectional surveys.

## INTRODUCTION

The global progress made in reducing the prevalence of cigarette smoking^1^ may have led to another challenge for tobacco control. The tobacco industry’s fear of losing its profitability and political influence has led to diversification of products^2,3^. Successful new products include electronic nicotine delivery systems (ENDS), which were first developed in 2003. The estimated global market for ENDS expanded from $3 billion^4^ to $10 billion^5^ from 2013 to 2015. However, the growth in the use of ENDS subsequently decreased due to their failure to deliver nicotine rapidly and provoke the sensations cigarettes provide^6,7^. Subsequently, heated tobacco products (HTP), which are similar to cigarettes, emerged in 2014 and were marketed with a ‘reduced harm’ statement^8^. The tobacco industry expects HTPs to rehabilitate its reputation; the chief executive officer of Philip Morris International (PMI) stated these products are the ‘greatest growth opportunity in the years to come’^9^. In June 2015, Pax Labs (currently JUUL Labs) launched JUUL, a USB-shaped ENDS: this re-ignited ENDS use, with its market share among ENDS increasing from <5% to >70% in 2 years^10^. Later models of ENDS, including JUUL, are capable of delivering an equal or greater amount of nicotine compared to conventional cigarettes^11^. These non cigarette tobacco or nicotine products (NCTNPs) may confuse the public and renormalize tobacco use due to their claims of reduced harm, designer looks, and approval or certification by a governmental organization^3^. Each product also has the potential to dilute tobacco control measures aimed at marginalizing the use and industry of tobacco products.

Determining whether a new NCTNP is a threat or opportunity to tobacco control requires identifying behavioral transitions between tobacco or nicotine products. Some advocate ENDS as a means to reduce tobacco-related harm, others express concern over the possibility of renormalization of tobacco use^12^. With some exceptions, the evidence generally indicates that ENDS^13^ and HTPs^14^ contain fewer or lower levels of toxicants (found in cigarette smoke) compared to combustible cigarettes, but also expose users to other chemicals not found in cigarette smoke. Those chemicals include aerosolized propylene glycol, vegetable glycerin, chemical flavorings as well as protonated nicotine (also known as nicotine salts)^15^. The health effects of chronic exposure to such chemicals are unknown. Further, reduced health risks cannot be substantiated without considering the behavioral patterns of tobacco or nicotine product use. One public health concern regarding NCTNP use behaviors is whether these products induce a gateway effect, leading users to start using a more harmful tobacco or nicotine product to obtain nicotine more effectively^16^. Furthermore, studies on gateway effects from ENDS to cigarettes have been criticized by ENDS advocates for not considering the gateway out of cigarette smoking to ENDS. For the claims of the ENDS advocates to be true, empirical evidence must show that behavioral transitions occur from ever cigarette smoking to ENDS, and non-current use of cigarette smoking^17^.

Behavioral patterns of use of these products should be explored first and foremost among adolescents, who are curious about novel and high-technology products^18^, more vulnerable to nicotine induced harm^19^, and are also at the forefront of social change such as the movement for a tobacco-free world^20^. The primary aim of tobacco control policies is to deter initiation^21^, so tobacco or nicotine use among adolescents should be investigated in terms of product experimentation (i.e. ever use). Not all adolescents who experiment become established users, but experimentation during adolescence is one of the strongest predictors of tobacco use in adulthood^22^. Once the sequential order of ever use status is identified, progression to regular use needs to be assessed.

Clarifying behavioral transitions requires longitudinal data. Timely monitoring of tobacco use is essential, as delaying data collection can result in a large number of users becoming addicted with catastrophic consequences. Monitoring is particularly important for newly introduced NCTNPs, because these are quickly adopted by younger generations^18^; however, a long time is required to collect longitudinal data, that include the use of both tobacco/nicotine products already on the market, and products just introduced. An alternative to a time-consuming longitudinal study is a cross-sectional cohort study^23^, which involves a cross-sectional cohort and retrospective assessment of exposure and outcomes. By constructing a retrospective cohort using recall data of a nationally representative sample of Korean adolescents, we examined whether use of ENDS leads to cigarette smoking initiation, and whether those who started ENDS then progressed to cigarettes and use cigarettes regularly. In addition, we examined whether those who experimented first with cigarette smoking then progressed to ENDS, eventually stop from being current cigarette smokers.

## METHODS

### Data source

We used data from the 14th (2018) Korea Youth Risk Behavior Survey (KYRBS), which explores healthrelated behaviors of Korean adolescents (school grades: 7–12). Details of the survey have been published previously^24^. Briefly, KYRBS is a nationally representative cross-sectional survey with multi-stage cluster sampling conducted in June 2018. The school participation rate was 100%, and 95.6% of students completed an anonymous, web-based survey. In total, 60040 adolescents completed the survey; 244 were subsequently excluded for missing values of grade at initiation of cigarette (n=205) and ENDS (n=38) use, and paternal educational attainment (n=1), so 59796 individuals were used in the analysis. This study was exempt from review by the Seoul National University Institutional Review Board.

### Study design

We used a cross-sectional cohort design^23^ to generate longitudinal data on adolescent tobacco or nicotine use. This design yields similar estimates to a cohort study, with similar validity conditions to traditional retrospective studies^23^. The same approach was used by Mayet et al.^25^ to assess the transition between cigarette smoking and cannabis use. We used a single cross-sectional sample (n=59796). Because the participants in the cross-sectional survey were asked about their grade at first experimentation with cigarettes and ENDS, we were able to construct a retrospective cohort based on the subjects’ recall of the grades during which they first used cigarettes and/ or ENDS. As participants’ use-states in the previous grade and current grade were identified using the constructed cohort, adolescents were assumed to be followed for 4 months from the end of their previous grade (end of February 2018) to the time of survey administration (June 2018).

### Measures

The products assessed were cigarettes and ENDS. Ever users of each product were defined as participants who responded ‘yes’ to any of the following questions: ‘Have you ever smoked a cigarette, even one puff?’ or ‘Have you ever used an electronic cigarette?’.

For never users of each product, the states at both baseline and follow-up were coded as ‘never’. For ever users of cigarettes and/or ENDS, the follow-up states were coded as ‘ever’. The baseline state for cigarettes and ENDS was assessed by inquiring about the grade at the time of initiation of these products. Ever users were asked: ‘When was the first time you smoked a cigarette, even just one or two puffs?’ and/ or ‘When was the first time you used an electronic cigarette?’. Possible responses were: ‘Before entering elementary school’, or in the range ‘grade 1’ to ‘grade 12’. The data also provided current school grade (at June 2018) of each adolescent, enabling the time of initiation of each product to be classified into ‘current grade’ and ‘previous grade or earlier’. The baseline state for cigarettes and/or ENDS was coded ‘ever’ for initiation at the previous grade or earlier and ‘never’ for initiation at the current grade. In South Korea, the school year begins on the first day of March and ends on the last day of February in the subsequent year.

The self-reported use states at baseline and during the follow-up were as follows: (1) never use, i.e. never use of both cigarettes and ENDS; (2) cigarette only use, i.e. ever cigarette use and never use of ENDS; (3) ENDS only use, i.e. ever ENDS use and never use of cigarettes; and (4) cigarette and ENDS use, i.e. ever use of both cigarettes and ENDS.

**Figure 1:**
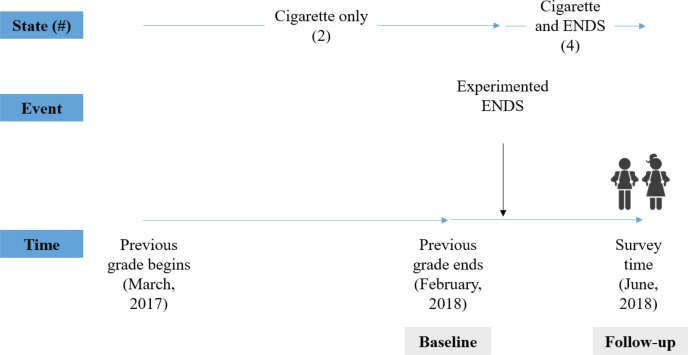
Example classification of tobacco/nicotine use

Figure 1 shows an example of tobacco or nicotine use classification. For an adolescent who reported cigarette initiation at the ‘previous or earlier grade’, and first experimented with ENDS during the ‘current grade’, the use state at the baseline was (2) cigarette only use, and the state at the end of the follow-up was (4) cigarette and ENDS use.

Current users of cigarettes and ENDS were identified as adolescents responding: ‘1–2’, ‘3–5’, ‘6– 9’, ‘10–19’, ‘20–29’ days or ‘everyday’ to ‘During the past 30 days, how many days did you smoke cigarettes, even one cigarette?’ and ‘During the past 30 days, how many days did you use e-cigarettes?’, respectively.

The covariates used were sex (male/female), grade at the baseline (6–11), ever alcohol use at the baseline year, and paternal education level. The questions: ‘Have you ever had at least one drink of alcohol’ and ‘When was the first time you had at least one drink of alcohol?’ (responses: ‘before entering elementary school’ or ‘grade 1’ to ‘grade 12’) were used to assess ever use of alcohol at baseline. Paternal education level was assumed to be invariant during the observation period, and was evaluated by the question: ‘What is your father’s educational level?’ (responses: ‘middle school or lower’, ‘graduated high school’, graduated university or higher’, and ‘don't know’). Responses of ‘middle school or lower’ and ‘graduated high school’ were coded as high school or lower; ‘graduated university or higher’ was recoded as college or higher. Those who answered ‘don't know’ for paternal education level and adolescents whose father was absent were recoded as unknown/not applicable (father absent).

### Statistical analyses

Participants’ characteristics were assessed by use states at baseline. A multistate Markov model was constructed to estimate transitions between two of the four use states; it assumes that the future state of the process is dependent on its present state and independent of its past state^26^. Participants could remain at the current state or add use of one or more product. Only one-way transitions were allowed, as use states were measured in terms of initiation of use.

**Figure 2:**
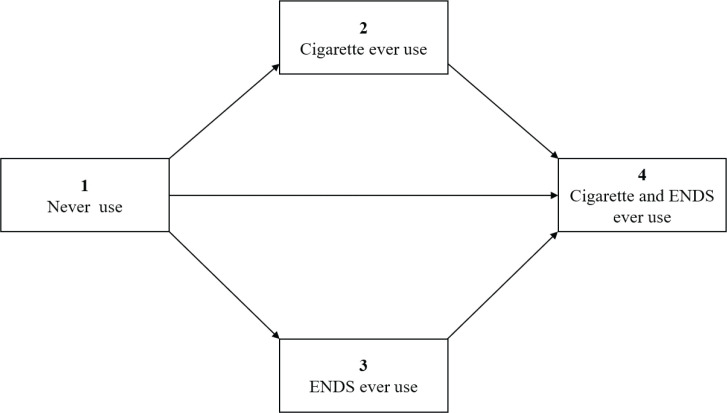
Possible transitions of tobacco/nicotine use states

The transitions are governed by a transition intensity (TI) matrix^26^, which is the instantaneous risk of moving from state i to j, as shown in Figure 2. The TI coefficients were calculated by the maximum likelihood method, and the corresponding transition intensity ratios (TIRs) and transition probabilities (TPs) were computed. The TIR is the ratio of the TIs of two sets of transition states and can be interpreted as the relative risk. The TP_ij_, from state i to j, reflects the conditional probability of remaining in or moving to a state within a given time t.

To address the first aim (ENDS as a gateway to cigarettes) the risk of never users initiating cigarette use (1→2) was compared with the risk of ENDS ever users initiating cigarette use (3→4). Next, the proportion of current cigarette smokers was calculated and compared for never users who initiated cigarette smoking, and ENDS ever users who initiated cigarette smoking. Regarding the second aim (cigarette use to ENDS use then non-regular cigarette use), the risk of never users initiating ENDS (1→3) versus cigarette ever users initiating ENDS use (2→4) was analyzed. The proportions of current users of cigarettes and/ or ENDS were then calculated among those who initiated first ENDS then cigarettes. In the Markov model, covariates were added one by one to the null model to assess its fit (Supplementary file, Table S1) and their impacts are shown in Supplementary file, Table S2. The Markov model was fitted using the msm package^27^ in R.

## RESULTS

### Participants

Table 1 lists the characteristics of participants. At baseline, most participants were never users (86.1%), followed by users of cigarettes only (7.5%), users of cigarettes and ENDS (5.4%), and ENDS only ever users (1.0%). Among the participants, 48% were females. With the exception of never use (51.9%), males comprised the majority of adolescents in all use states. The grade distribution among never users was fairly uniform. For the rest of the use states, a large proportion of adolescents was in higher grades. The distribution of paternal education level was 53.7% college or higher, 25.1% high school or lower, and 21.2% unknown or not applicable. Among never users, 28.9% had ever used alcohol. The majority of participants in the cigarette only (74.9%), ENDS only (67.4%), and cigarette and ENDS (89.0%) states were ever alcohol users.

**Table 1:** Participant characteristics by tobacco or nicotine product use state at baseline, February 2018

*Characteristics*	*Total*	*Use state at baseline (state number )*			
		*Never use (1)*	*Cigarette only (2)*	*ENDS only (3)*	*Cigarette and ENDS (4)*
	*n (%)*	*n (%)*	*n (%)*	*n (%)*	*n (%)*
**Total**	59796 (100.0)	51905 (86.1)	4399 (7.5)	552 (1.0)	2940 (5.4)
**Female sex**	29490 (48.0)	27475 (51.9)	1355 (28.9)	119 (19.1)	541 (17.2)
**Grade**					
6	9805 (14.5)	9545 (16.4)	202 (3.9)	30 (4.1)	28 (0.8)
7	10039 (15.7)	9397 (17.0)	428 (9.3)	56 (9.0)	158 (4.8)
8	10224 (16.2)	9038 (16.7)	755 (15.5)	85 (14.3)	346 (10.7)
9	9219 (15.9)	7773 (15.6)	795 (17.6)	104 (18.9)	547 (17.9)
10	9997 (17.8)	8041 (16.6)	988 (22.6)	143 (26.5)	825 (27.6)
11	10512 (19.9)	8111 (17.8)	1231 (31.0)	134 (27.2)	1036 (38.2)
**Paternal education level**					
College or higher	31112 (53.7)	27614 (55.0)	1961 (46.1)	260 (48.7)	1277 (44.4)
High school or lower	15200 (25.1)	12635 (23.9)	1396 (31.3)	159 (28.8)	1010 (34.4)
Unknown/NA	13484 (21.2)	11656 (21.1)	1042 (22.6)	133 (22.5)	653 (21.2)
**Ever alcohol use**	20906 (36.0)	14630 (28.9)	3294 (74.9)	366 (67.4)	2616 (89.0)

ENDS: electronic nicotine delivery systems. NA: not applicable.

### Markov model

**Figure 3:**
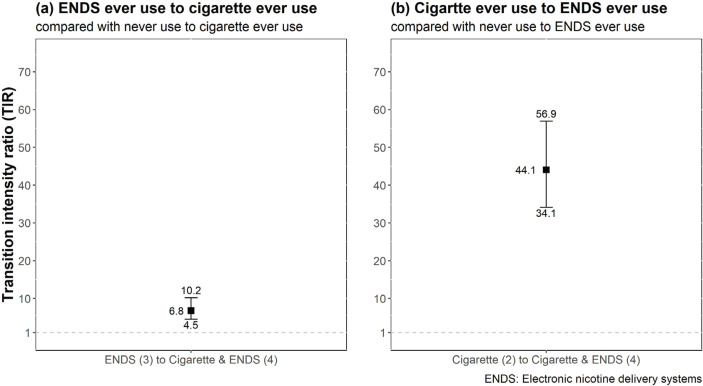
Ratio of the transition intensities between paired tobacco/nicotine use states

The model that incorporated all of the covariates showed the best fit (Supplementary file, Table S1). Table 2 lists the frequencies of remaining in and moving to another state. The TPs (Table 2) and TIRs (Figure 3) are provided from the full model, in which the estimates were calculated at the mean of all covariates. Most of the never users remained never users after four months (TP_11_=98.17%, 95% CI: 98.04–98.29%), while few never users initiated use of cigarettes (TP_12_=1.39%, 95% CI: 1.29–1.49%), ENDS (TP_13_=0.21%, 95% CI: 0.17–0.26%), or cigarettes and ENDS simultaneously (TP_14_=0.23%, 95% CI: 0.18– 0.29%). The majority of adolescents who used a single product remained in that state after 4 months, users of cigarettes only (TP_22_=90.49%, 95% CI: 89.04–91.75%) and users of ENDS only (TP_33_=90.48%, 95% CI: 86.15–93.43%).

**Table 2:** Frequencies of state transitions and transition probabilities (TPs) of tobacco or nicotine product use

*Baseline state (state number)*	*End state (state number)*			
	*Never use (1)*	*Cigarette only (2)*	*ENDS only (3)*	*Cigarette and ENDS (4)*
*Frequency of state transitions*	*n (%)*	*n (%)*	*n (%)*	*n (%)*
Never (1)	50836 (85.0)	760 (1.3)	144 (0.2)	165 (0.3)
Cigarette only (2)	-	3883 (6.5)	-	516 (0.9)
ENDS only (3)	-	-	494 (0.8)	58 (0.1)
Cigarette and ENDS (4)	-	-	-	2940 (4.9)
Transition probabilities (%)*	*TP (95% CI)*	*TP (95% CI)*	*TP (95% CI)*	*TP (95% CI)*
Never (1)	98.17 (98.04–98.29)	1.39 (1.29–1.49)	0.21 (0.17–0.26)	0.23 (0.18–0.29)
Cigarette only (2)	-	90.49	- (89.04–91.75)	9.51 (8.25–10.96)
ENDS only (3)	-	-	90.48 (86.15–93.43)	9.52 (6.57–13.85)
Cigarette and ENDS (4)	-	-	-	100.0

TP: transitional probabilities. ENDS: electronic nicotine delivery systems. *Obtained from the Markov model adjusted for sex, grade, ever alcohol use, and paternal education level.

Figure 3(a) provides the TIRs for ENDS ever users initiating cigarette use. Compared with never use, ever use of ENDS only (TP_34_=9.52%, 95% CI: 6.57–15.83%) was associated with a 7-fold increased risk of initiating cigarette use (TIR=6.8, 95% CI: 4.5– 10.2). Figure 3(b) presents the transitions from ever cigarette use to ENDS use, compared with never use to ENDS use. The proportion of those initiating ENDS use, among ever users of cigarettes, was similar to that of cigarette ever users initiating ENDS (TP_24_=9.51%, 95% CI: 8.25–10.96%). The risk of transitioning from cigarette ever use to ENDS ever use was 44-fold that of transitioning from never use to ENDS ever use (TIR=44.1, 95% CI: 34.1–56.9).

### Current use of cigarettes/ENDS

Table 3 lists the prevalence of current cigarette and/ or ENDS use by ever use state transition. Among those who were never users at baseline and who had experimented with cigarette use at follow-up, 35.8% (95% CI: 32.1–39.5%) were current cigarette smokers. By contrast, the prevalence of cigarette smoking was higher (43.1%, 95% CI: 30.0–56.2%) among those who started ENDS first and subsequently used cigarettes. More than one-quarter of adolescents who started using cigarettes and subsequently progressed to ENDS remained current cigarette smokers (27.8%, 95% CI: 23.6–32.0%). Some of these adolescents were current ENDS users (7.2%, 95% CI: 4.9–9.6%). Almost half of these adolescents were current users of both cigarettes and ENDS (46.4%, 95% CI: 42.1– 51.1%). In total, 74.4% of adolescents who moved from the (2) cigarette only state to the (4) cigarette and ENDS state were current users of cigarettes or both cigarettes and ENDS.

**Table 3:** Prevalence of current cigarette and/or ENDS use by different ever use state transitions

*Transition of ever use states (state number)*	*Total*	*Current use*					
		*Cigarette only*		*ENDS only*		*Cigarette and ENDS*	
	*n*	*n*	*% (95% CI)*	*n*	*% (95% CI)*	*n*	*% (95% CI)*
Total	1643	474		92	306		
Never (1) →Cigarette only (2)	760	278	35.8 (32.1–39.5)	-	-	-	-
Never (1) → ENDS only (3)	144	-	-	39	25.9 (19.2–32.6)	-	-
Never (1) →Cigarette and ENDS (4)	165	28	18.6 (11.0–26.2)	11	5.3 (2.2–8.4)	61	37.2 (29.5–45.0)
Cigarette only (2) → Cigarette and ENDS (4)	516	144	27.8 (23.6–32.0)	37	7.2 (4.9–9.6)	236	46.6 (42.1–51.1)
ENDS only (3) →Cigarette and ENDS (4)	58	24	43.1 (30.0–56.2)	5	10.1 (2.1–18.1)	9	15.7 (6.6–24.8)

ENDS: electronic nicotine delivery systems.

## DISCUSSION

Using multistate Markov modelling of a nationally representative sample of Korean adolescents, we evaluated the longitudinal transitions of tobacco or nicotine use and obtained three important findings. First, ever use of ENDS led to subsequent use of cigarettes. Second, initiating ENDS use after cigarette ever use was common. In addition, a large proportion of adolescents who initiate ENDS then cigarettes and *vice versa* remained as current cigarette smokers.

Transitioning from use of ENDS to cigarettes is reportedly frequent^28,29^. ENDS are sometimes considered to reduce the harm of tobacco use^30^; but this is disputed by our findings on the actual behavior of adolescents. We found that the risk of initiating cigarette use increases markedly after experimentation with ENDS, and a previous study reported that the majority of adolescent ENDS users were also cigarette users^31^. The results of our study showed the prevalence of current cigarette smoking was 1.2fold higher for those who started cigarette smoking subsequent to ENDS use, compared to those who had not experimented with ENDS. Additionally, the risk of ENDS initiation was higher after ever cigarette use. High risk of cigarette ever users initiating ENDS use is in agreement with a previous report that NCTNP use is associated with previous use of tobacco products^32^. This finding may reflect the future of tobacco use, and can be used to develop regulations for NCTNPs. In South Korea, where JUUL was introduced in May 2019, the prevalence of cigarette use could increase rapidly. Despite such finding, NCTNPs are typically subject to less strict control measures than cigarettes^16^. To reduce initiation of tobacco or nicotine use, control measures for NCTNPs must be at the same level as those for cigarettes and include integrated measures of price increases, mass-media advertising, smoke-free policies, school programs, reducing access to tobacco products, and marginalizing the tobacco industry^21^.

A continuum of regular cigarette smoking was found for adolescents who progressed from cigarette ever use to ENDS ever use. The positioning of ENDS as a gateway out of cigarettes is erroneous, because 28% of adolescents remained users of conventional cigarettes. Worse, nearly half of adolescents who progressed from cigarette ever use to ENDS ever use are current users of both cigarettes and ENDS. Our results suggest that using ENDS does not assist in quitting cigarette smoking, and it may expose adolescents to greater adverse health effects as they engage in the use of multiple products. Rather than tobacco/nicotine products with claims of reduced harm, a robust and comprehensive tobacco control program is required to help adolescents quit cigarette smoking.

### Strengths and limitations

Several limitations of the study must be noted. Because of the cross-sectional cohort design of the study, it is possible that participants who dropped out of school and therefore left the study cohort, have different use characteristics. However, these adolescents accounted for <2% of the total cohort^33^. Second, use of self-reported measures may have led to underestimation of the prevalence of use of tobacco or nicotine products. Third, we assessed only the use of ENDS to examine its association with cigarettes. However, other NCTNPs, such as HTPs, may also be strongly associated with cigarette smoking. This study suggests an important methodological aspect of monitoring of newly introduced NCTNPs. The design adopted in this study, i.e. construction of a retrospective cohort based on the recall data of a cross-sectional sample, has several limitations, such as recall bias, compared to a conventional longitudinal design. However, by employing the proposed design, we can provide rough estimates of behavioral transitions related to tobacco or nicotine products. To use such a design, cross-sectional surveys of tobacco or nicotine use must include questions on the timing of initiation of each product. The KYRBS, the source of the data used in this study, asks participants about the timing of experimentation with cigarettes and ENDS but not HTPs. Thus, participants must be also asked about the timing of their first experience with HTPs. Further, questions on the timing of onset of daily use would provide information on the time of initiation of regular use of tobacco/nicotine products.

## CONCLUSIONS

We constructed a retrospective cohort with crosssectional data on the transition between cigarette and ENDS. Our findings demonstrate that ever use of ENDS increases the likelihood of initiation of cigarette smoking. Moreover, a significant proportion of adolescents who progressed from cigarette ever use to ENDS ever use remained current cigarettes smokers or cigarette and ENDS dual users. Although NCTNPs are regulated less stringently than cigarettes, our findings support the need for strong measures to curb initiation of their use and more research on how they are used in the real world^18^. This will require monitoring the use of all types of tobacco or nicotine products^34^. Combined with measures to assess the timing of initiation of use of tobacco products, a retrospective longitudinal study can be constructed from cross-sectional data.
