# Supplementary material for: Longitudinal transitions of cigarettes and electronic nicotine delivery systems among adolescents: Construction of a retrospective cohort using recall data from a cross-sectional sample
Source: Tob Induc Dis. 2020 Nov 5;18:92. doi: 10.18332/tid/128488 (PMC7656743; doi:10.18332/tid/128488)
Supplement: Supplementary file 1 [file TID-18-92-s1.pdf]

SUPPLEMENTARY MATERIAL

Results

Markov model

Table S1 Log-likelihood ratios for different covariate combinations

Model	-2 log-likelihood ratios	Δ	df	p-value
No covariate	15681			
Sex	15569	112.4	5	<.0001
Sex, grade	15533	36.2	5	<.0001
Sex, grade, paternal education	15491	41.8	5	<.0001
Sex, grade, paternal education, ever alcohol use	15172	318.8	5	<.0001

Each column of the Δ -2log-likelihood ratio represent the test against the model immediately above

Table S2 Hazard ratios and 95% confidence intervals of covariate impacts on the transition processes

Transition (in state numbers)	Sex (ref=female)	Grade	Paternal education (ref=Unknown/NA)		Ever alcohol use
			High school or lower	College or higher	
	HR (95% CI)	HR (95% CI)	HR (95% CI)	HR (95% CI)	HR (95% CI)
N (1) → C (2)	**1.2 (1.0-1.3)**	1.0 (0.9-1.0)	1.0 (0.9-1.3)	0.9 (0.7-1.0)	**3.0 (2.6-3.5)**
N (1) → E (3)	**3.5 (2.4-5.0)**	1.1 (1.0-1.2)	1.0 (0.6-1.5)	0.7 (0.4-1.0)	**3.7 (2.6-5.2)**
N (1) → CE (4)	**4.9 (2.3-10.4)**	0.9 (0.8-1.1)	1.0 (0.6-1.8)	**0.4 (0.2-0.8)**	**2.2 (1.3-3.7)**
C (2) → CE (4)	**1.4 (1.1-1.7)**	**0.9 (0.9-1.0)**	1.1 (0.9-1.4)	0.9 (0.7-1.1)	**1.6 (1.3-2.0)**
E (3) → EC (4)	1.9 (0.9-4.0)	**0.7 (0.6-0.9)**	0.9 (0.4-2.0)	1.2 (0.6-2.3)	1.1 (0.7-2.0)

N (1): Never use; C (2): cigarette only; E (3): ENDS only; CE or EC (4): cigarette and ENDS;
Significance at p<0.05 are bolded.

© 2020 Kang H. and Cho S.
